# Lymphoplasmacytic lymphoma associated with diffuse large B-cell lymphoma: Progression or divergent evolution?

**DOI:** 10.1371/journal.pone.0241634

**Published:** 2020-11-12

**Authors:** Macarena Boiza-Sánchez, Rebeca Manso, Olga Balagué, Cristina Chamizo, Elham Askari, Rocío Nieves Salgado, Carlos Blas-López, Elena Aguirregoicoa-García, Javier Menárguez, Carlos Santonja, Magdalena Adrados, Miguel Ángel Limeres-González, Miguel Ángel Piris, Socorro María Rodríguez-Pinilla

**Affiliations:** 1 Pathology Department, Hospital Universitario Fundación Jiménez Díaz, Madrid, Spain; 2 Pathology Department, Hospital Clinic, Barcelona, Spain; 3 Hematology Department, Hospital Universitario, Madrid, Spain; 4 Cytogenetics Service, Hospital Universitario Fundación Jiménez Día, Madrid, Spain; 5 Pathology Department, Hospital Universitario Gregorio Marañón, Madrid, Spain; 6 Pathology Department, Hospital Universitario de la Princesa, Madrid, Spain; 7 Pathology Department, Hospital Universitario de Gran Canaria “Doctor. Negrín”, Las Palmas de Gran Canaria, Spain; European Institute of Oncology, ITALY

## Abstract

**Aim:**

Lymphoplasmacytic lymphoma (LPL) is an indolent mature B-cell-neoplasm with involvement of the bone marrow. At least 90% of LPLs carry *MYD88-L265P* mutation and some of them (~10%) transform into diffuse large B-cell-lymphoma (DLBCL).

**Material and methods:**

Over the past 15 years we have collected 7 cases where the both LPL and DLBCL were diagnosed in the same patient. Clinical records, analytical data and histopathological specimens were reviewed. FISH studies on paraffin-embedded tissue for *MYC*, *BCL2* and *BCL6* genes were performed, as well as *MYD88-L265P* mutation and *IGH* rearrangement analysis by PCR. A mutational study was done by massive next generation sequencing (NGS).

**Results:**

There were 4 women and 3 men between 36–91 years of age. Diagnoses were made simultaneously in 4 patients. In two cases the LPL appeared before the DLBCL and in the remaining case the high-grade component was discovered 5 years before the LPL. In 6 cases both samples shared the *MYD88-L265P* mutation. *IGH* rearrangement analysis showed overlapping features in two of 6 cases tested. Mutational study was evaluable in three cases for both samples showing shared and divergent mutations.

**Conclusions:**

These data suggest different mechanisms of DLBCL development in LPL patients.

## Introduction

Lymphoplasmacytic lymphoma (LPL) is a low-grade B-cell lymphoma (LGBL) composed of B cells at different differentiation stages, consisting of small lymphocytes, plasmacytoid lymphocytes, and plasma cells, not fulfilling criteria for any other small B-cell lymphoid neoplasm. Bone marrow (BM) is usually involved followed in frequency by lymph nodes and spleen. Most patients are clinically diagnosed with Waldenström macroglobulinemia (WM), which is defined as LPL with 10% or more BM involvement and an immunoglobulin M (IgM) monoclonal gammopathy of any concentration. The cause of WM remains unknown although a role for genetic, immune-related, and environmental factors has been suggested [1–3]. Next-generation sequencing studies in WM have identified multiple recurring somatic mutations, including *MYD88-L265P* (up to 97%), *CXCR4* (30%-40%), *ARID1A* (17%), and *CD79B* (8%-15%) [3, 4]. Although *MYD88-L265P* mutation seemed to be very characteristic of this disease, it has also been described in other LGBLs with lower frequency, namely in MZL (6%-10%) and chronic lymphocytic leukemia/small lymphocytic lymphoma (CLL/SLL) (3% to 8%) [5].

More than 10% of WMs transform into more aggressive disorders, such as diffuse large B-cell lymphoma (DLBCL) and their prognosis appears to be worse than that for patients with de novo DLBCL, with survival from the time of transformation of about 2 years [6, 7]. Transformation to DLBCL can occur at any time point along the course of WM, i.e. at diagnosis, before treatment, during therapy and even 20 years after the initial diagnosis. Interestingly, this secondary DLBCL can be either clonally or not clonally related to the WM neoplasm [6–8]. These facts make the distinction between progression of a low-grade B-cell lymphoma and the development of a second primary aggressive tumor sometimes challenging.

We identified 7 patients diagnosed with both LPL and DLBCL. Clinical data and molecular features of all these cases were heterogeneous. Interestingly, all cases but one showed the *MYD88-L265P* mutation in both neoplastic components. The significance of this finding in the development of both LPL and DLBCL is discussed.

## Material and methods

### Patient samples

Seven formalin-fixed, paraffin-embedded (FFPE) samples were retrieved from the Biobank of Fundación Jiménez Díaz (Madrid, Spain). All patients gave informed consent to be included in this study, being their data anonymized. Diagnoses were established according to the WHO classification [9]. Patients’ diagnosis, medical management and follow up was done between years 2000–2019.

The Comité de Ética de la Investigación de la Fundación Jiménez Díaz, CEIm-FJD approved the study “Linfomas agresivos, análisis clínico y genómico para una medicina de precisión (B2017/BMD-3778. LINFOMAS CM. CI: PIC75-018)” in which this study is included.

### DNA and RNA extraction

We extracted genomic DNA (DNAg) and RNA of tumoral FFPE samples using a RecoverAll^™^ Multi-Sample RNA/DNA (Invitrogen, Carlsbad, CA, USA) in accordance with the manufacturer’s protocol. DNAs and RNAs were quantified with Qubit^®^ (Invitrogen, Carlsbad, CA, USA).

### PCR for IgH gene rearrangement

PCR was performed to analyze the clonal expansion of B-cells. DNA was extracted from paraffin sections and B-cell clonal expansion was detected by PCR for the immunoglobulin heavy (*IGH*, VH—JH and DH—JH) chain gene rearrangement [10] (S1 Table). Appropriate positive and negative controls were included in all experiments. Clonality was assayed following well-established recommendations [11].

### qPCR detection of MYD88-L265P mutation

To identify the presence of the *MYD88* mutation, we used the primers described by Jiménez *et al*. [12] and quantitative PCR (qPCR) based in allelic discrimination (S2 Table). Triplicates were performed on a Light Cycler 480 Real-Time PCR System (Roche, Basilea, Suiza). Assays were run in 384-well plates in a reaction volume of 10 μl, using 50 ng of genomic DNA (gDNA), 5 μl of Light Cycler 480 Probes Master, 0.15 μl primer (20μM, wild type or mutated) and 0.1μl probe (20μM). The reaction mixture was incubated at 95°C for 10 min, followed by 45 cycles of amplification at 95°C for 10 s and 60°C for 30 s. The data were analyzed with software Light Cycler 480 SW 1.5 (Roche) using the ΔCp method. Cp values were analyzed and showed a standard deviation minor of 0.25. Cases with a Cp value >20 were considered wild type.

### Mutation with the TruSeq custom amplicon

Dual-strand sequencing eliminates false-positive C-T mutations that can arise from deamination events during formalin fixation. The probes for this custom panel were designed with DesignStudio (Illumina, San Diego, CA, USA) and consisted of 1399 amplicons with an average size of 175 bp and a cumulative targeted region of 140 kb [13]. Polymorphisms were avoided in the design of the primers. The target genes are listed in S3 Table.

Target enrichment was performed in FFPET-extracted DNA according to manufacturer’s instructions (TruSeq Amplicon—Cancer Panel Library Preparation Guide; January 2017; Illumina). The total amount of input DNA ranged from 30 to 100ng. After library preparation, indexing and bead purification, the libraries (two libraries per sample, one per strand) were quantified by Qubit^®^ (Invitrogen, Carlsbad, CA, USA) and then normalized with beads and pooled for sequencing. The pooled libraries were sequenced with a Miseq Reagent Kit V2 (paired-end, 2x151) on a MiSeq instrument (Illumina, San Diego, CA, USA), as described in the manufacturer’s protocol.

### Mutation with Oncomine Comprehensive Assay (OCA)

The Oncomine Comprehensive Assay, OCA (Ion Torrent, ThermoFisher Scientific, Waltham, Massachusetts, USA) was used to validate common data. The DNA/RNA libraries were performed according to manufacturer’s instructions (OncomineTM Comprehensive Assay v3 Guide; ThermoFisher Scientific, Waltham, Massachusetts, USA). DNA libraries were generated from 20 ng of DNA using the Ion AmpliSeq Library Kit 2.0 (Life Technologies, Carlsbad, CA, USA) and the OCP AmpliSeq panel according to the manufacturer’s instructions with barcode incorporation. RNA libraries were generated from 15 ng of RNA using the Ion AmpliSeq RNA Library Kit. Templates for DNA and RNA libraries were prepared using the Ion AmpliSeq^™^ Library Preparation the Ion 540^™^ Chef system (Life Technologies, Carlsbad, CA, USA) on the Ion Chef according to the manufacturer’s instructions. Sequencing of multiplexed templates was performed using the Ion Torrent S5 on Ion 540 chips (Life Technologies, Carlsbad, CA, USA) according to the manufacturer’s instructions.

### Bioinformatic analysis

The sequences were aligned with the reference genome NCBI Build 37 (UCSC hg19). Variants were then identified by the Variant Caller algorithm and the annotation of variants was performed with the Ion Reporter (Life Technologies, Carlsbad, CA, USA) and Variant Interpreter (Illumina, San Diego, CA, USA). We mapped reads to the hg19 reference genome using the Integrated Genome Viewer (IGV v2.3; Broad Institute, Cambridge, MA, USA). The Catalogue of Somatic Mutations in Cancer (COSMIC), the National Center for Biotechnology ClinVar and cBioPortal for Cancer Genomics database were checked to identify pathogenetic changes. In addition, the variants were analysed with two mutational functional prediction programs (sift and polyphen-2).

### Immunohistochemistry

FFPE sections from both LPL and DLBCL samples of all patients were stained by the EndVision method with a heat-induced antigen-retrieval step for a panel of 13 different antibodies (S4 Table). Reactive tonsil tissue was included as a control. The primary antibodies were omitted to provide negative controls.

## Results

Over the past 15 years 7 patients were identified with diagnoses of both LPL and DLBCL. Here we have compared the clinical, histological and molecular data of both tumours in this series of patients in an effort to unveil the mechanism of tumour progression.

### Clinical features

There were 4 women and 3 men, with ages between 36 and 91 years (mean 61 years).

Four patients (57%) showed B symptoms (fever, night sweats and weight loss) as early manifestations, and 28% experienced bone pain. Less frequent symptoms were fatigue and dysphagia or hemorrhagic stroke (each 14%). One patient showed multiple asymptomatic enlarged lymph nodes.

Monoclonal paraproteinemia (IgM) was found at diagnosis in all of them and cryoglobulinemia was detected in one case (Table 1).

**Table 1:** Clinical data of our series of patients (DOD: Dead of disease; DOC: Dead of other cause; CR: Complete response; DLBCL: Diffuse large B-cell-lymphoma; LPL: Lymphoplasmacytic lymphoma).

	CASE 1	CASE 2	CASE 3	CASE 4	CASE 5	CASE 6	CASE 7
**Clinical features**	B symptoms Bone pain	B symptoms	Asymptomatic adenopathies	Hemorrhagic stroke	B symptoms Bone pain	Fatigue B symptoms	Dysphagia Nasal congestion
**Blood/urine test**	IgM 1066 mg/dl M-protein IgM κ Hb 9,1 g/dl	IgM 1940 mg/dl M-protein IgM λ Hb 13,9 g/dl	IgM 325 mg/dl M-protein IgM κ Hb 8,9 g/dl Cryoglobulinemia	IgM 1780 mg/dl M-protein IgM κ Hb 12,8 g/dl	IgM 3063 mg/dl M-protein IgM κ Hb 8,5 g/dl	IgM 2025 mg/dl M-protein IgM κ Bence-Jones κ Hb 8,1 g/dl	IgM 1654 mg/dl M-protein IgM κ Hb 13,7 g/dL
**First diagnosis Location/ Tumour Type**	Bone marrow LPL	Adenopathies + Bone Marrow non-GC DLBCL + LPL, respectively	Multiple adenopathies non-GC DLBCL	CNS non-GC DLBCL	Pelvic adenopathies LPL	Adenopathies+BM Pancreatic & renal involvement non-GC DLBCL	Pharyngeal mass Adenopathies non-GC DLBCL
**Treatment**	CDR x6	R-CHOP	NA	NA	NA	R-CHOP x3	NA
**Second diagnosis Location/Tumour type**	Mandibular mass + Adenopathies DLBCL ABC	CNS (relapse) non-GC DLBCL	Bone marrow LPL	Adenopathies + Bone marrow LPL	Bone marrow non-GC DLBCL + LPL	Cavum mass + Bone marrow LPL	Bone marrow LPL
**2° Treatment**	R-CHOP x6	MTX/RITUXIMAB	R-CHOP + RCD	MTX/RITUXIMAB	R-CHOP x1	RITUX/BENDA	R-CHOP x6
**Current state**	DLBCL: CR LPL: relapse BM	DLBCL: CR M-protein persists DOC (craneal trauma)	DLBCL: CR LPL: minimum marrow infiltrate	DLBCL: CR LPL: PR (stable disease)	DOC (severe reaction to Rituximab)	DLBCL: CR LPL: PR (stable disease)	DOD (clinical progression)

In one case the DLBCL preceded the LPL (case 6), with an interval of 5 years. In four cases both lymphoma types were simultaneously diagnosed (cases 2, 3, 4 and 7), although in case 4; the DLBCL was diagnosed two months earlier than the LPL lymphoma. In another two cases the diagnosis of LPL preceded the diagnosis of DLBCL after 3 and 1 year, respectively (case 1 and 5).

Lymphoplasmacytic lymphoma was diagnosed in bone marrow (4 cases), lymph node (2 cases) or cavum (one case), although all 7 cases showed bone marrow infiltration. In the histological findings, one case had predominance of plasmacytoid cellularity (with numerous Dutcher and Russell bodies) and another case showed a predominance of mature B cells. The other cases had mixed cellularity.

Four of the DLBCL cases were diagnosed on lymph nodes, one in central nervous system, one in bone marrow and one in Waldeyer’s ring. Additionally, one patient showed pancreatic and renal involvement.

All patients received treatment. R-CHOP was the most frequently applied chemotherapy regimen (86%), usually with completion of 6 cycles. One patient died after the first cycle due to a severe reaction to Rituximab. Two patients received R-CHOP in combination with either Bendamustine-Rituximab or Methotrexate-Rituximab. Only one patient received exclusively chemotherapy with Methotrexate-Rituximab.

The outcome was favorable in 4 cases (57%), with complete response of the high-grade component and stable disease of the LPL component (minimum marrow infiltrate). One of these cases (Case 1) suffered a bone marrow relapse three years after stabilization of the LPL and remitted after Ibrutinib treatment. Three patients died; one from traumatic brain injury, one due to Rituximab toxicity, and one from disease progression (Case 7); the latter was the only case with no *MYD88-L265P* mutation shared by both samples.

### Immunohistochemical features

**Fig 1:**
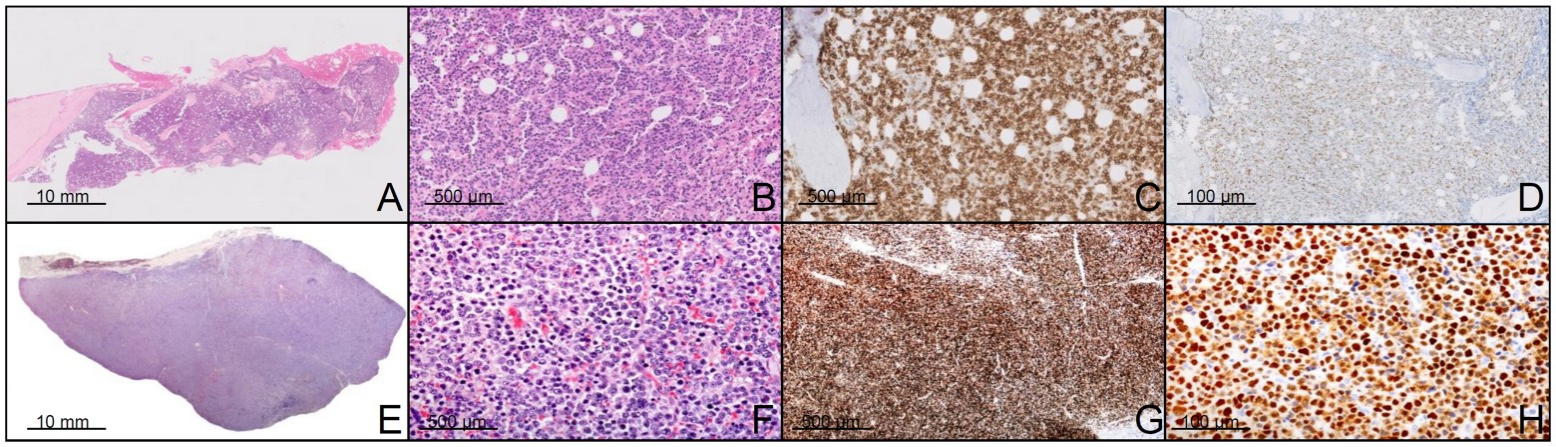
Case 1 showing p53 expression in both LPL and DLBCL component. A-B. H-E staining of the bone marrow biopsy at different magnification showing a diffuse interstitial infiltration of lymphoid and lymphoplasmocytoid cells expressing CD20 (C) and P53 (D). In E (H-E staining) a diffuse proliferation of neoplastic cells effacing the lymph node architecture is seen. At higher magnification (F) these cells showed inmunoblastic or plasmablastic morphology (H-E staining), high proliferation index (G) with KI67 and p53 positivity (H). Scale bars of figures A-E is 10 mm, figures B-F is 500μm and figures D-H is 100μm.

Two of the LPL cases expressed p53 (Cases 1 and 5) and one was CD10 positive (Case 6) (Fig 1). All cases expressed IgM with kappa light chain restriction, with the exception of Case 2 that was lambda.

**Fig 2:**
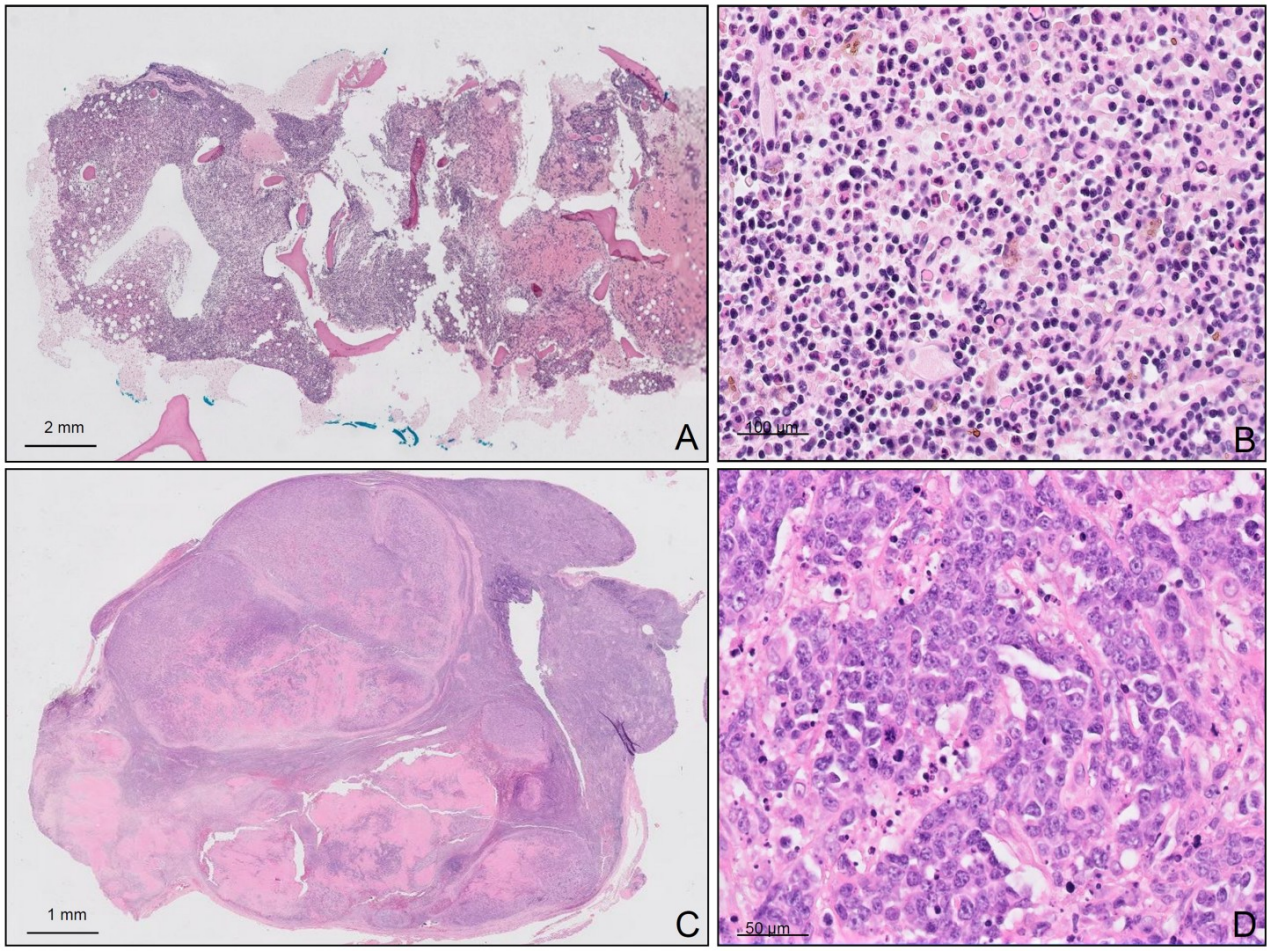
Another LPL case evolving into DLBCL is shown. In figure A (H-E) and B (H-E) bone marrow biopsy with lymphoplasmacytic infiltration is seen. Cytoplasmic inmunoglobulins displacing the nuclei to the periphery of the cell are clearly seen (B). In C (H-E), effacement of lymph node architecture by the proliferation of large cells with inmunoblastic morphology is seen (D) (H-E). Scale bars of figures A is 2 mm, figure Bis 100μm, figure C is 1 mm and figure D is 50 μm.

According to Hans’s algorithm, all DLBCL cases were considered of non-germinal center B-cell phenotype (non-GCB) (Fig 2).

There were CD10 negative, MUM1 positive. None of the cases were EBV (EBER) positive. Three of them were p53-positive (Cases 1, 5 and 7). Cases 1 and 5 developed two and one years after the diagnosis of LPL, respectively and p53 was also present in the LPL component from the beginning of the disease.

### Molecular characteristics

Six out of these seven patients (86%) shared the *MYD88-L265P* mutation, detected by q-PCR, in both neoplastic components (LPL and DLBCL). Case number 7 showed *MYD88-L265P* mutation in the LPL component while the DLBCL was wild-type (Table 2).

**Table 2:** NGS results and IgH rearrangement analysis in all cases.

Case n°	Lymphoma type	NGS	IgH rearrangement			Relationship of LPL and DLBCL
		(all panels)	FR1	FR2	FR3	
**1**	LPL	*MYD88-L265P* (1%)	333	264	124, 126	Progression
		*ARID1A-V2041A* (45%)				
	DLBCL	*MYD88-L265P* (90%)	333	264	115, 124	
		*ARID1A-V2041A* (31%)				
		ARID1A-Q1818* (30%)				
**2**	LPL	*MYD88-L265P* (7%)	339, 340	273	139, 140	Common clonal origin + Divergent differentiaton or Syncronous not related
		*CXCR4-S342fs*				
	DLBCL	*MYD88-L265P* (32%)	NA	261	121	
		PIM1-Q218* (16%)				
**3**	LPL	*MYD88-L265P* (39%)	334, 335	Polyclonal	126, 127	Progression
	DLBCL	*MYD88-L265P* (1%)	334, 335	267	118, 126, 127	
**4**	LPL	*MYD88-L265P* (39%)	NA	266, 267	133	Common clonal origin + Divergent differentiaton or Syncronous not related
		*NOTCH1-P2415del* (3.7%)				
	DLBCL	*MYD88-L265P* (16%)	NA	248	105	
		*CD79B-A30V (7%)*				
		*PIM1-G71V (9%)*				
		*PIM1-G91A (9%)*				
		*PIM1-S188R (10%)*				
		*PIM1-D199E (10%)*				
		*PIM1-I266V (9%)*				
**5**	LPL	*MYD88-L265P* (18%)	NA	NA	NA	Common clonal origin
	DLBCL	*MYD88-L265P* (49%)	NA	NA	Polyclonal	
		BAP1-Q684* (9%)				
**6**	LPL	*MYD88-L265P* (34%)	NA	279	130	Common clonal origin + Divergent differentiaton or Syncronous not related
		*TBL1XR1-PIK3CA* (Fusion)				
	DLBCL	*MYD88-L265P* (17%)	NA	Pseudoclonal	Pseudoclonal	
**7**	LPL	*MYD88-L265P* (39%)	NA	267	126, 133, 134	Unrelated
		*MYC-P74S* (47%)				
	DLBCL	WT	NA	256	Pseudoclonal	

In boldface type, shared mutation between low- and high-grade components (DLBCL: diffuse large B-cell-lymphoma; LPL: Lymphoplasmacytic lymphoma; NGS: Next-Generation Sequencing; WT: Wild-Type).

No alterations of *BCL2*, *BCL6* or *MYC* genes were found in any case by FISH studies performed on paraffin-embedded-tissue.

*IGH* PCR studies (using Biomed primers) showed identical clonal peaks in both tumoral components in two cases, and different in four cases. In the remaining case (Case 5) the PCR technique was not evaluable.

The clonal peak differed in size between both components in three of the four cases diagnosed synchronously. Regarding cases initially diagnosed as LPL and developing a DLBCL during the course of the disease, one case showed the same clonal rearrangement in both components (Case 1), while in the other one the study was not informative (Case 5).

Next generation sequencing (NGS) was performed for both samples of every patient, using two different technical approaches and two different gene panels. In three patients at least one of the panels could be analyzed in both tumoral samples of the same patient, being in only two patients both methods informative. In the other cases one or both panels could be analyzed in at least one of the samples. In all LPL samples at least one of the two methods employed identified an additional alteration to *MYD88-L265P* mutation, while we obtained additional mutational data in just 3 DLBCL samples.

Apart from the *MYD88-L265P* mutation, other mutations were shared by the two tumoral components of the same patient in only one case (Case 1). Both the LPL and the DLBCL components of Case 1 showed *ARID1A-V2041A* mutation. Interestingly, a similar clonal rearrangement in both components was found. The DLBCL developed two years after the initial LPL diagnosis and showed an additional mutation in ARID1A-Q1818* gene.

Other genes mutated in the LPL component, in one case each, were *CXCR4* (Case 2), *NOTCH 1* (Case 4), *BAP1* (Case 5) or *MYC* (Case 7). Interestingly, a novel fusion between *TBL1XR1-PIK3CA* genes in a LPL case was also found (Case 6).

DLBCL cases showed additional mutations in *ARID1A*, *PIM1* and *CD79B* genes. Different mutations of *PIM1* gene were found in two different cases. No mutations of the *TP53* gene were found in any case. Allele frequencies around 50% were considered as polymorphisms.

## Discussion

Progression of LPL into DLBCL has been estimated to occur in about 10% of the cases. We describe 7 patients diagnosed with both LPL and DLBCL, where clonal identity is supported by the presence of *MYD88-L265P* mutation (Cases 1–6), identical *IGH* rearrangements (Cases 1 and 3) or simultaneous presence in both components of additional mutational data (Case 1). Notwithstanding this common clonal origin, our series hints at divergent differentiation, as supported by the differences in the serial sample analysis of *IGH*, (3 cases showed different *IGH* rearrangements) and newly acquired somatic mutations.

**Fig 3:**
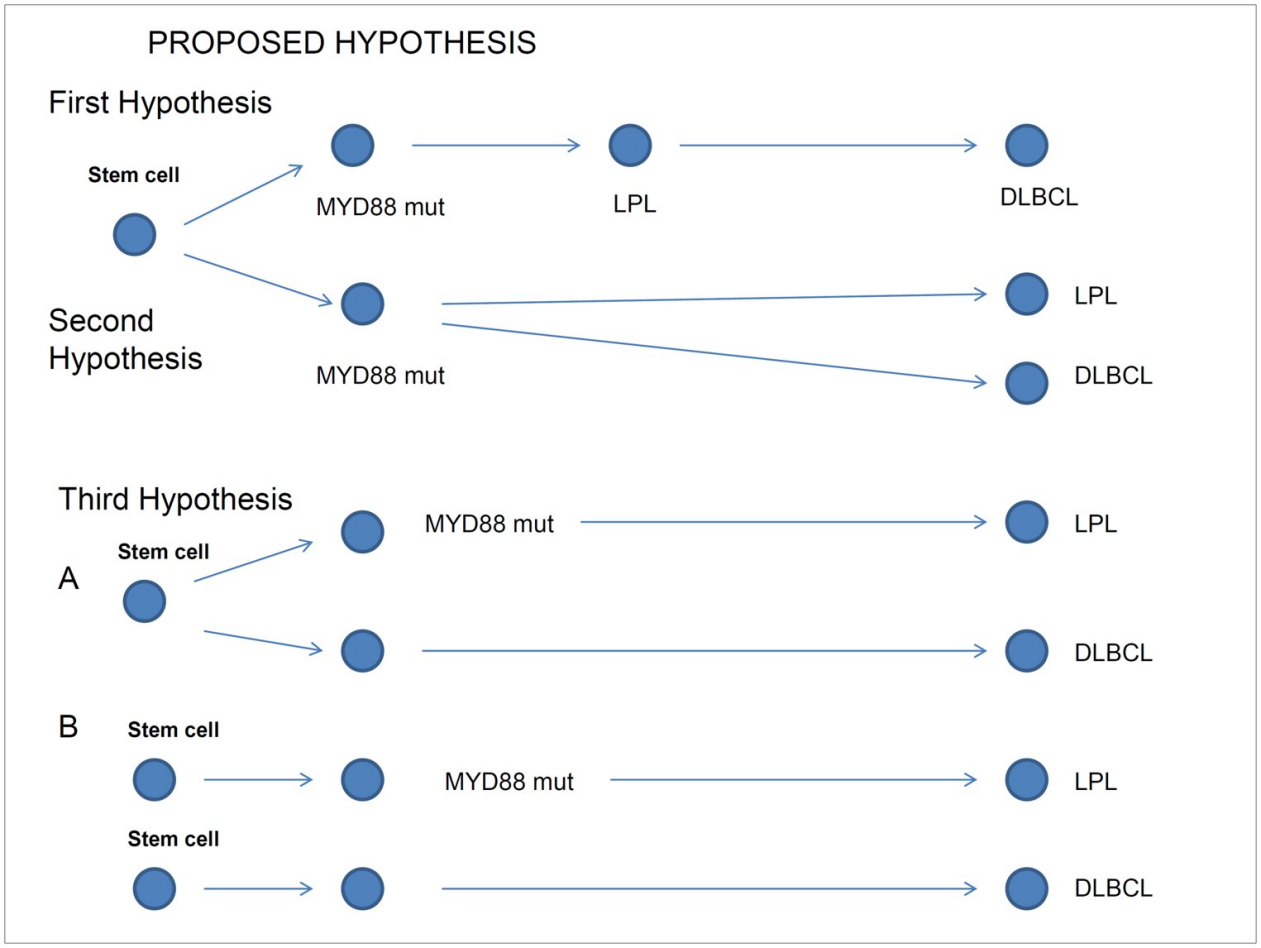
Hypothesis of DLBCL development in patients with LPL. First Hypothesis: Sequential mutation adquisition from LPL to DLBCL. Second hypothesis: Common origin with divergent evolution of either LPL and DLBCL neoplasms. Third hypothesis: A. Different lymphomas from a common stem cell or B. Different lymphomas from different stem cells.

Based on the one by one analysis of the cases we propose that there are at least three possible mechanisms of DLBCL development in LPL patients (Fig 3).

In our hands the largest subgroup of patients consisted of those with shared *MYD88* mutations in both lymphoma types, but harbored different *IGH* clonal peaks. In these cases a divergent clonal evolution from a common precursor neoplastic cell is proposed. Nevertheless, the appearance of two independent synchronous neoplasms cannot be definitively ruled out.

*MYD88* mutation has been found in healthy individuals as well as in peripheral blood mononuclear cells of patients with CNS lymphoma [14, 15], suggesting that this alteration might take place before *IGH* rearrangement. This data suggest that both tumors arose from cells harboring the *MYD88* mutation that further suffered either class switch recombination, somatic hypermutation or ongoing *IGH* mutations, leading to two partially-different independent tumoral clones [16]. *MYD88-L265P* mutation has been considered a driver mutation in the development of WM, and in most cases of MGUS that evolve into WM [4].

Recently, biclonality in WM patients has been documented [17, 18] and at least in 20% of WM patients two clonotypic sequences have been detected [19]. These data suggest that in some cases WM, rather than consisting of a monoclonal population, harbors two clonal populations evolved from a common ancestor [17, 18]. In the present study, this could explain that although LPL and DLBCL neoplastic cells of the same patient shared a *MYD88* mutation, they had different *IGH* rearrangement. Moreover, in most of our cases further somatic mutations in other genes were found exclusively in either the LPL or the DLBCL components. These findings, as well as previously reported data support our first hypothesis [20, 21]. Nevertheless, there are no biological models proving that *MYD88* mutation takes place before *IGH* rearrangement.

Durot *et al*. [22] analyzed 77 patients with transformed LPL and found that most DLBCL showed extranodal involvement (91% of their cases). The most commonly involved extranodal sites were the bone marrow (18%), followed by bone (14%), spleen (14%) and central nervous system (13%). Moreover, most cases (82%) were of non-GC phenotype, like in our series, where all 7 DLBCL cases displayed a non-GC phenotype. It has been classically considered that the clonal lymphoplasmacytic cell phenotype in WM corresponds to the late stage of B-cell differentiation and is derived from IgM-producing memory B cells that have undergone somatic hypermutation, but not isotype switching [16]. In general, it is worth mentioning that most ABC-DLBCL has not undergone class switching, and ABC-DLBCL express IgM more frequently than GCB-DLBCL [23, 24]. Moreover, it is well known that cases with *MYD88-L265P* mutations are enriched in the ABC subtype [23, 24] and represent about 24% of the DLBCL cases. This percentage is high in DLBCL cases occurring in immune-privileged sites such as CNS (70%) and testes (74%), as well as in cutaneous leg-type DLBCL (54%), which are frequently of the ABC subtype [23, 24]. One of our DLBCL cases developed in CNS few months after the diagnosis of LPL in lymph node and bone marrow. This DLBCL also presented further somatic mutations not present in the LPL component and also frequently found in primary CNS DLBCL, such as *PIM1* and *CD79B* [15, 25]. On the other hand, Jimenez C *et al*. [26] identified *CD79B* and *PIM1* as one as the most frequently mutated genes in the transformation event in LPL cases or in the DLBCL component. Whether these two neoplasms took place as independent events although coincidentally in time or are originated from a common precursor cell harboring the *MYD88* gene mutation cannot be elucidated. Furthermore, two cases in our series (50%) arose in lymph nodes (cases 2 and 6). The *MYD88* mutation has been described in 17% of nodal DLBCL [27]. Moreover, both sporadic and familial cases of LPL have been associated with an increased risk of developing other hematological diseases, especially DLBCL [2, 28]. So, again, a coincidental, synchronous development of two independent hematological processes (LPL and DLBCL) cannot be easily be ruled out.

Interestingly, in some LPL cases other somatic mutations such as *CXCR4* (Case 2), *NOTCH 1* (Case 4), *BAP1* (Case 5) or *MYC* (Case 7) and the novel fusion between *TBL1XR1-PIK3CA* genes were also found (Case 6). These alterations in the DLBCL component of our cases could not be studied due to technical problems.

CXCR4 mutations are detected in 30–40% of LPL cases and in 9% of IgM MGUS [29, 30]. There are over 30 different mutations in the C-terminus of the *CXCR4* gene identified in WM patients. The most common type is S338X nonsense mutation that causes increased activation of the Akt, ERK, BTK pathways resulting in enhanced migration, adhesion, growth and survival of cells in WM [29, 30]. Mutation in *CXCR4* gene also confers primary resistance to Ibrutinib treatment [31, 32]. As far as we know, neither *NOTCH1*, *BAP1* or *MYC* gene mutations have been reported so far in LPL. Moreover, *BAP1* has not been previously found to be mutated in B-cell lymphomas.

In only two patients the DLBCL developed after the initial diagnosis of LPL, suggesting a progression /transformation of the initial LPL (cases 1 and 5). Interestingly, in these two cases intense p53 expression was observed in both components, but no *P53* gene mutations could be identified in the sequencing study. *P53* mutation has been reported at diagnosis in 7.5% of LPL cases, being at lower frequency in IgM MGUS and associated to poor outcome [30]. It is highly correlated to deletion of 17p and to cases with higher number of genetic alterations [33]. Interestingly, Jiménez C *et al*. [26] identified p53 gene mutations by whole-exome sequencing studies in two patients at transformation or at progression, but not by the time of disease transformation of this last patient. They suggested that the transformed final clone did not originate in the intermediate subclone responsible from progression, but from a previous minor subclone that only grew after progression. Furthermore, they did not attribute p53 gene a key role in the transformation process.

In Case 1 the LPL and the DLBCL neoplastic cells shared both *IGH* rearrangements and further somatic mutations. *ARID1A-V2041A* mutation was shared by both tumoral matched samples (LPL and DLBCL) while a further mutation in the same gene (ARID1A-Q1818*) was exclusively found in the DLBCL component. *ARID1A* is a gene implicated in chromatin organization, being reported in 17% of LPL patients. These cases could represent a classic progression phenomenon, confirming some of the results previously published by Jiménez C *et al*. [26] after whole exome sequencing, showing the simultaneous presence of *MYD88-L265P* mutation in serial samples from MW progressing to LBCL. Based on their data, the authors suggest a common progenitor cell for both neoplasms, although following a branching model in the evolution process. Interestingly, all the patients they analyzed were initially diagnosed with LPL, with later development of the DLBCL after a median of 52 months. In our hands those cases suffered transformation after 35 and 12 months (median of 23.5 months), respectively.

On the other hand, in case 7 no *MYD88* gene mutation could be identified in the DLBCL component, although it was present in the LPL clone diagnosed at the same time. Moreover, they showed different clonal *IGH* rearrangements and high intensity of p53 expression exclusively in the DLBCL component. Nevertheless, these data suggest the occurrence of a synchronous lymphoma with independent clonal origin, somehow similar to the case recently described by Talaulikar *et al*. [8]. On the other hand, lack of sensitivity of the employed techniques may have hindered the detection of other common molecular alterations in this case.

It is remarkable that in 4 out of 7 of our cases both LPL and DLBCL were diagnosed at the same time (cases 2, 3, 4 and 7). This frequency (57%) is much higher than previously reported (17%) [34] which could be due to reference bias, since we receive specimens from all over the country. Three of these cases showed different *IGH* rearrangements in either LPL or DLBCL counterpart samples. This data are in agreement with the case reported by Shimizu S *et al*. [35] who suggested a different clonal origin for both diseases after sequencing the CDR3 region.

Classically, it is believed that LPL without *MYD88* mutation progresses to DLBCL with a higher probability than LPL cases with *MYD88* mutation [36, 37]. Nevertheless, in our hands wild type *MYD88* LPL cases are exceptional (only two documented cases in our archive) and none of them progressed into a more aggressive lymphoma.

In conclusion, our data point to the various progression scenarios in patients with metachronous or simultaneous occurrence of LPL and DLBCL. Some cases are consistent with tumoral progression, others (case 7) seem to represent a second malignancy, and while a significant group supports that a *MYD88*-mutated common progenitor gave rise to different clones characterized by divergent differentiation.

## Supporting information

S1 Table List of primers of the *IGH* gene rearrangement. (DOCX)

S2 Table List of primers of the *MYD88* gene. (DOCX)

S3 Table List of probes of the custom B-cell lymphoma panel. (DOCX)

S4 Table List of proteins analyzed in this series of patients. (DOCX)
