# Supplementary material for: Lymphoplasmacytic lymphoma associated with diffuse large B-cell lymphoma: Progression or divergent evolution?
Source: PLoS One. 2020 Nov 12;15(11):e0241634. doi: 10.1371/journal.pone.0241634 (PMC7661053; doi:10.1371/journal.pone.0241634)
Supplement: S1 Table — (DOCX) [file pone.0241634.s001.docx]

**S1 Table. List of primers of the *IGH* gene rearrangement.**

| **Primer Name** | **Primer Sequence (5' to 3')** |
| --- | --- |
| VH1-FR1 | GGCCTCAGTGAAGGTCTCCTGCAAG |
| VH2-FR1 | GTCTGGTCCTACGCTGGTGAAACCC |
| VH3-FR1 | CTGGGGGGTCCCTGAGACTCTCCTG |
| VH4-FR1 | CTTCGGAGACCCTGTCCCTCACCTG |
| VH5-FR1 | CGGGGAGTCTCTGAAGATCTCCTGT |
| VH6-FR1 | TCGCAGACCCTCTCACTCACCTGTG |
| VH1-FR2 | CTGGGTGCGACAGGCCCCTGGACAA |
| VH2-FR2 | TGGATCCGTCAGCCCCCAGGGAAGG |
| VH3-FR2 | GGTCCGCCAGGCTCCAGGGAA |
| VH4-FR2 | TGGATCCGCCAGCCCCCAGGGAAGG |
| VH5-FR2 | GGGTGCGCCAGATGCCCGGGAAAGG |
| VH6-FR2 | TGGATCAGGCAGTCCCCATCGAGAG |
| VH7-FR2 | TTGGGTGCGACAGGCCCCTGGACAA |
| VH1-FR3 | TGGAGCTGAGCAGCCTGAGATCTGA |
| VH2-FR3 | CAATGACCAACATGGACCCTGTGGA |
| VH3-FR3 | TCTGCAAATGAACAGCCTGAGAGCC |
| VH4-FR3 | GAGCTCTGTGACCGCCGCGGACACG |
| VH5-FR3 | CAGCACCGCCTACCTGCAGTGGAGC |
| VH6-FR3 | GTTCTCCCTGCAGCTGAACTCTGTG |
| VH7-FR3 | CAGCACGGCATATCTGCAGATCAG |
| JH-Consensus | [6FAM]CTTACCTGAGGAGACGGTGACC |
