# Supplementary material for: Lymphoplasmacytic lymphoma associated with diffuse large B-cell lymphoma: Progression or divergent evolution?
Source: PLoS One. 2020 Nov 12;15(11):e0241634. doi: 10.1371/journal.pone.0241634 (PMC7661053; doi:10.1371/journal.pone.0241634)
Supplement: S2 Table — (DOCX) [file pone.0241634.s002.docx]

**S2 Table. List of primers of the *MYD88* gene.**

| **Primer Name** | **Primer Sequence (5' to 3')** |
| --- | --- |
| MYD88L265P RV wt | CCT TGT ACT TGA TGG GGA TCA |
| MYD88L265P RV mut | CCT TGT ACT TGA TGG GGA TGG |
| MYD88L265P FW wt | ACT TAG ATG GGG GAT GGC TG |
| MYD88L265P probe | (6FAM)TTG AAG ACT GGG CTT GTC CCA CC(TAM) |
