# Supplementary material for: Lymphoplasmacytic lymphoma associated with diffuse large B-cell lymphoma: Progression or divergent evolution?
Source: PLoS One. 2020 Nov 12;15(11):e0241634. doi: 10.1371/journal.pone.0241634 (PMC7661053; doi:10.1371/journal.pone.0241634)
Supplement: S3 Table — (DOCX) [file pone.0241634.s003.docx]

**S3 Table. List of probes of the custom B-cell lymphoma panel.**

| **Genes** | **Region** | **Cromosome** | **Start** | **Stop** |
| --- | --- | --- | --- | --- |
| ARID1A | Coding region |  |  |  |
| ATM | Coding region |  |  |  |
| ATP6AP1 | HotSpot | X | 153663637 | 153664238 |
| ATP6V1B2 | HotSpot | 8 | 20072478 | 20074805 |
| B2M | Coding region |  |  |  |
| BCL2 | Coding region |  |  |  |
| BRAF | Coding region |  |  |  |
| BTK | Coding region |  |  |  |
| CARD11 | Coding region |  |  |  |
| CCND3 | Coding region |  |  |  |
| CD79A | Coding region |  |  |  |
| CD79B | Coding region |  |  |  |
| CREBBP | Coding region |  |  |  |
| CXCR4 | Coding region |  |  |  |
| EP300 | Coding region |  |  |  |
| EZH2 | Coding region |  |  |  |
| FAT2 | Coding region |  |  |  |
| FOXO1 | Coding region |  |  |  |
| ID3 | HotSpot | 1 | 23885454 | 23886078 |
| MEF2B | Coding region |  |  |  |
| MLL2 | Coding region |  |  |  |
| MYC | Coding region |  |  |  |
| MYD88 | Coding region |  |  |  |
| NOTCH1 | Coding region |  |  |  |
| NOTCH2 | Coding region |  |  |  |
| PIM1 | Coding region |  |  |  |
| PLCG2 | Coding region |  |  |  |
| PRDM1 | Coding region |  |  |  |
| PTPN1 | Coding region |  |  |  |
| RRAGC | HotSpot | 1 | 39322619 | 39325195 |
| SGK1 | Coding region |  |  |  |
| SMARCA4 | Coding region |  |  |  |
| STAT3 | Coding region |  |  |  |
| STAT6 | Coding region |  |  |  |
| TCF3 | Coding region |  |  |  |
| TNFAIP3 | Coding region |  |  |  |
| TNFRSF14 | Coding region |  |  |  |
| TP53 | Coding region |  |  |  |
