# Supplementary material for: Lymphoplasmacytic lymphoma associated with diffuse large B-cell lymphoma: Progression or divergent evolution?
Source: PLoS One. 2020 Nov 12;15(11):e0241634. doi: 10.1371/journal.pone.0241634 (PMC7661053; doi:10.1371/journal.pone.0241634)
Supplement: S4 Table — (DOCX) [file pone.0241634.s004.docx]

**S4 Table. List of proteins analyzed in this series of patients.**

| **ANTIBODY** | **CLONE** | **MANUFACTURED** | **REFERENCE** | **CONTROL** | **RECUPERATION** | **AMPLIFICATION** | **DILUTION/INCUBATION TIME** | **STAINER** | **TEMPERATURE** |
| --- | --- | --- | --- | --- | --- | --- | --- | --- | --- |
| **CD20cy** | L26 | DAKO | GA604 | Amígdala | High | Flex | RTU 20 | OMNIS | 4º |
| **CD79** | JBC117 | DAKO | IR621 | Amígdala | Low | Flex | RTU 8 | OMNIS | 4º |
| **CD 3-L** | Polyclonal | DAKO | GA503 | Amígdala | Low | Flex+ | RTU 10 | OMNIS | 4º |
| **CD10-L** | 56C6 | DAKO | GA648 | AMIGDALA | Low | Flex+ | RTU 45 | OMNIS | 4º |
| **BCL2** | 124 | DAKO | IR614 | Amígdala | High | Flex+ | RTU 20 | OMNIS | 4º |
| **BCL6** | PG-B6p | DAKO | GA625 | Amígdala | High | Flex+ | RTU 25 | OMNIS | 4º |
| **MUM1** | MUM1p | DAKO | IR644 | AMIGDALA | High | Flex + | RTU 30 | OMNIS | 4º |
| **C-MYC** | Y69 | VENTANA | 790-4628 | Amígdala | CC1 | AMPL | ROCHE 32 | BENCHMARK | 4º |
| **P53** | DO-7 | DAKO | GA616 | AMIGDALA | CC1 | Flex | RTU 15 | OMNIS | 4º |
| **Ki67 -L** | MIB-1 | DAKO | GA506 | AMIGDALA | low | Flex | RTU 20 | OMNIS | 4º |
| **IgM-L** | Polyclonal | DAKO | IR513 | AMIGDALA | Low | Flex | RTU 4 | OMNIS | 4º |
| **KAPPA** | Polyclonal | DAKO | GA506 | AMIGDALA | High | Flex | RTU 1/5 8 | OMNIS | 4º |
| **LAMBDA** | Polyclonal | DAKO | GA507 | AMIGDALA | High | Flex | RTU 1/5 12 | OMNIS | 4º |
